# “COAGULATION”: a mnemonic device for treating coagulation disorders following traumatic brain injury—a narrative-based method in the intensive care unit

**DOI:** 10.3389/fpubh.2023.1309094

**Published:** 2023-12-06

**Authors:** Manuel Quintana-Diaz, Pasquale Anania, Raúl Juárez-Vela, Emmanuel Echaniz-Serrano, Clara Isabel Tejada-Garrido, Pilar Sanchez-Conde, Kapil Nanwani-Nanwani, Ainhoa Serrano-Lázaro, Pilar Marcos-Neira, María Gero-Escapa, Jorge García-Criado, Daniel Agustín Godoy

**Affiliations:** ^1^Department of Medicine, Faculty of Medicine, Autonomous University of Madrid, Madrid, Spain; ^2^Intensive Care Unit, La Paz University Hospital, Madrid, Spain; ^3^Institute for Health Research (idiPAZ), La Paz University Hospital, Madrid, Spain; ^4^Department of Neurosurgery, Ospedale Policlinico San Martino, Istituto di Ricovero eCura a Carattere Scientifico (IRCCS) for Oncology and Neuroscience, Genoa, Italy; ^5^Department of Nursing, University of La Rioja, Logroño, Spain; ^6^Health and Healthcare Research Group (GRUPAC), Faculty of Health Sciences, University of La Rioja, Logroño, Spain; ^7^Department of Nursing and Physiatry, Faculty of Health Sciences, University of Zaragoza, Zaragoza, Spain; ^8^Aragon Healthcare Service, Aragon, Zaragoza, Spain; ^9^Faculty of Medicine, University of Salamanca, Salamanca, Spain; ^10^Intensive Care Unit, Valencia University Clinical Hospital, Valencia, Spain; ^11^Intensive Care Unit, Germans Trias i Pujol University Hospital, Badalona, Spain; ^12^Intensive Care Unit, Burgos University Hospital, Burgos, Spain; ^13^Emergency Department, University Hospital of Salamanca, Salamanca, Spain; ^14^Critical Care Department, Neurointensive Care Unit, Sanatorio Pasteur, Catamarca, Argentina

**Keywords:** coagulopathy, diagnosis, traumatic brain injury, haemostasis, blood coagulation, anticoagulants, antithrombotic drugs

## Abstract

**Introduction:**

Coagulopathy associated with isolated traumatic brain injury (C-iTBI) is a frequent complication associated with poor outcomes, primarily due to its role in the development or progression of haemorrhagic brain lesions. The independent risk factors for its onset are age, severity of traumatic brain injury (TBI), volume of fluids administered during resuscitation, and pre-injury use of antithrombotic drugs. Although the pathophysiology of C-iTBI has not been fully elucidated, two distinct stages have been identified: an initial hypocoagulable phase that begins within the first 24 h, dominated by platelet dysfunction and hyperfibrinolysis, followed by a hypercoagulable state that generally starts 72 h after the trauma. The aim of this study was to design an acronym as a mnemonic device to provide clinicians with an auxiliary tool in the treatment of this complication.

**Methods:**

A narrative analysis was performed in which intensive care physicians were asked to list the key factors related to C-iTBI. The initial sample was comprised of 33 respondents. Respondents who were not physicians, not currently working in or with experience in coagulopathy were excluded. Interviews were conducted for a month until the sample was saturated. Each participant was asked a single question: Can you identify a factor associated with coagulopathy in patients with TBI? Factors identified by respondents were then submitted to a quality check based on published studies and proven evidence. Because all the factors identified had strong support in the literature, none was eliminated. An acronym was then developed to create the mnemonic device.

**Results and conclusion:**

Eleven factors were identified: cerebral computed tomography, oral anticoagulant & antiplatelet use, arterial blood pressure (Hypotension), goal-directed haemostatic therapy, use fluids cautiously, low calcium levels, anaemia-transfusion, temperature, international normalised ratio (INR), oral antithrombotic reversal, normal acid–base status, forming the acronym “Coagulation.” This acronym is a simple mnemonic device, easy to apply for anyone facing the challenge of treating patients of moderate or severe TBI on a daily basis.

## 1 Specific aims and theoretical grounds of the study

A narrative approach involves pluralism, relativism, and subjectivity. Despite advances in the care of neurocritically ill patients from the prehospital stage through rehabilitation, traumatic brain injury (TBI) continues to be a pathological entity associated with significant rates of functional disability and mortality (1). Narratives have classically been a mode of transmitting culture on macro-societal levels, and a way to create evidence. Irrespective of the mechanism of injury, TBI is understood to consist of two clearly defined processes (1–4). The primary injury depends on the type, location, and amount of energy absorbed by the structures that comprise the cranial cavity and the results of focal (intra or extra-axial haemorrhages) or diffuse (axonal damage) lesions. These lesions, in turn, trigger different and multiple neurotoxic cascades, most notably, the inflammatory and coagulation cascades that negatively influence haemodynamics, oxygenation, and cerebral energy metabolism (1–4). Secondary injury involves a series of phenomena that can originate within the skull (intracranial hypertension, cerebral hypoxia) or systemically (hypotension, hypoxemia, sodium, and glycaemic alterations) which combine to perpetuate or exacerbate the primary injury (1–4). While primary injuries are irreversible, secondary injuries can be prevented and corrected, and this is precisely one of the pillars upon which the modern management of severe TBI is based (1–4).

Normal haemostasis involves a delicate balance between mechanisms that promote bleeding and those that try to prevent it, a balance that can be disrupted following TBI. Coagulation disorders in severe isolated TBI (C-iTBI) are common and contribute to secondary damage, mainly due to their role in facilitating the development or progression of both ischemic and haemorrhagic lesions (5–10). TBI has increasingly come to affect older patients who often present with polypharmacy including antithrombotics and anticoagulants due to multiple comorbidities (11). The incidence of C-iTBI as reported in the literature averages 33%, with figures ranging from 7 to 90%. The disparities in results may be due to differences in study design, lack of uniform definition, varied cohorts, points of the parameters used to define it, concomitant presence of polytrauma, or the time it was analysed (5–10, 12). Nonetheless, based on these figures, we can posit that approximately one of three patients will develop C-iTBI, strongly suggesting its origin in the brain itself (7–10, 12).

The pathophysiology of C-iTBI has not yet been fully and accurately elucidated; however, current evidence indicates that it is a primary haemostasis disorder broadly characterised by an early hypocoagulable period, where platelet dysfunction and hyperfibrinolysis predominate, and a later prothrombotic stage, where the described mechanisms reverse their role (12). By platelet dysfunction, we understand the generic and non-specific term conventionally used to denote alterations in platelet physiology. In the context of trauma, this may either involve a decrease in number (thrombocytopenia) or function, i.e., alteration of one of the steps in which platelets are actively involved (adhesion, aggregation, and secretion). Such alterations may occur directly secondary to traumatic injury or as a consequence of pre-existing disease or prior use of antithrombotic medications. The incidence of platelet dysfunction ranges from less than 1% in mild TBI to >60% in severe TBI.

Predisposing factors identified for the development of C-iTBI include the severity of the trauma (GCS <9), age (> 75 years), previous fluid therapy (> 2 L), base excess (< −6), hypothermia, arterial hypotension, and prior use of anticoagulant or antiplatelet agents (12, 13). The presence of C-iTBI adds predictive power to validated prognostic scales (14, 15). C-iTBI is associated with poor outcomes in terms of functionality and mortality, which explains why this factor is a key therapeutic target to consider (7–10, 12–16). The real implications that early detection and correction of coagulation disorders have in the context of severe TBI remain unknown, however, they open up a wide range of future research possibilities.

Healthcare providers communicate the events of treating a difficult case through narratives. Narratives are also used to concretize a body of knowledge in specific contexts, as in the case of intensive care medicine. Stories encapsulate professional experiences to be shared with students in practise, or advanced professionals. In this study, we used the narrative experience of intensive care physicians to design a mnemonic device we called “Coagulation” to provide clinicians with an auxiliary tool in the treatment of C-iTBI.

## 2 Methods

To carry out this study based on narrative, the authors of the manuscript have selected the best possible evidence on the topic under study. Once selected, it was debated until the variables that were considered most important were obtained. Three rounds were carried out until the selection of variables began to be repeated and we considered that the selection was saturated, something that in qualitative methodology indicates the end of the selection process. The initial sample was comprised of 33 respondents. Respondents who were not physicians, not currently working in or with experience in coagulopathy were excluded. The process and the meeting to select variables were conducted for a month until the sample was saturated. In this meeting, all participants have been arranged to make the mnemonic rule more easily understandable.

Each participant was asked a single question: Can you identify a factor associated with coagulopathy in patients with TBI? The respondents identified specific concepts during the interviews which were then collected and mapped. Following the initial reading and validation, we consulted the literature to justify the factors identified.

Ultimately, we identified 11 factors.

The factors identified by the respondents were submitted to a quality check, based on the published literature and proven evidence. Because all the factors identified had strong support in the literature, none was eliminated.

## 3 Results

### 3.1 Factor 1: cerebral computed tomography was identified by participants as “Essential to the classification of severity and monitoring”

Cerebral computed tomography (CT) is the neuroimaging of choice during the acute phase of severe TBI (17–19). In addition to being widely available, it is cost-effective, has a short radiation exposure time and can even be performed on patients with prostheses or on mechanical ventilation (17–19). The CT scan allows for the simultaneous evaluation of other body regions and is an essential complementary tool to categorize TBI (17–19). It provides an objective assessment of the type and extension of lesions, their volume, location, and impact on intracranial anatomical structures (18–20). It is highly sensitive for the detection of blood collections (17, 18, 21). Finally, it yields pathophysiological information and has a proven predictive capacity for both intracranial hypertension and outcome (20, 22, 23).

**Figure 1:**
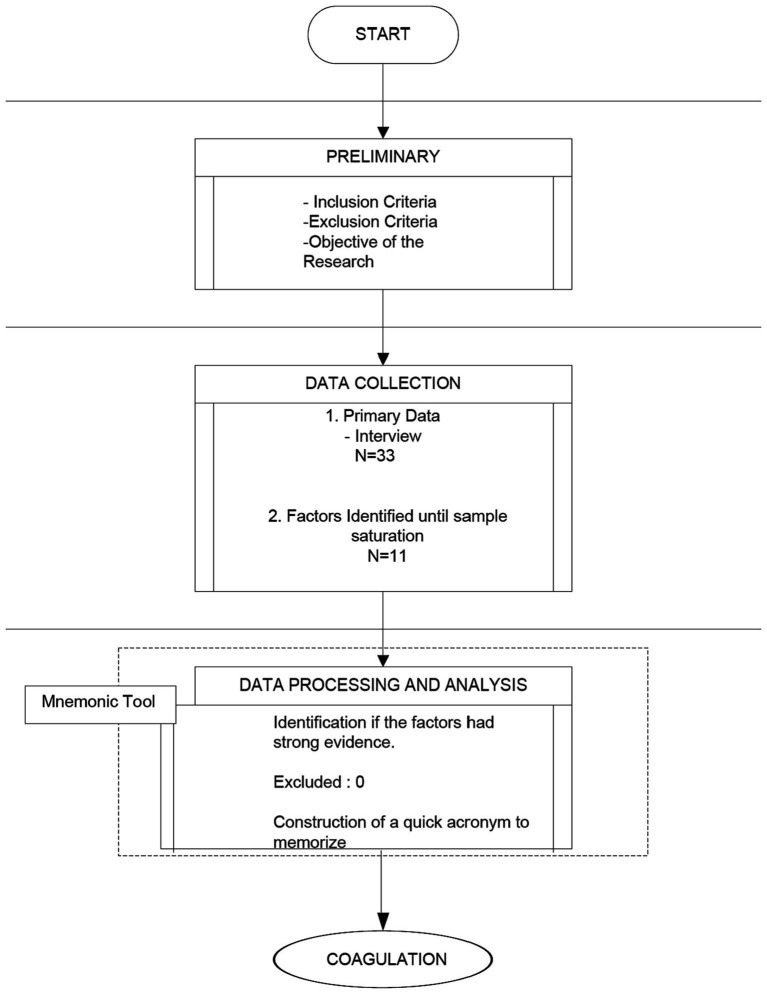
Flow chart.

Different CT scales have been developed and validated in the clinical field (20, 23–26). Its serial determination allows clinicians to monitor the progression of the broad spectrum of lesions following TBI (17–20, 22–26). Therefore, CT plays an important role in diagnosis, evaluation of lesion progression, and therapeutic decision-making in the context of C-iTBI (Figure 1).

### 3.2 Factor 2: oral anticoagulant and antiplatelet use was identified by participants as “High Alert”

The increase in life expectancy over the last decades has led to an increasingly older adult population with associated pathologies, mainly cardiac and cerebrovascular, that require the use of oral anticoagulants (OAC) or antiplatelet agents (APA). The global incidence of cerebral bleeding secondary to OAC/APA utilisation is 20% (12). Specifically, clinical studies have shown that the use of warfarin before iTBI doubles the possibility of poor outcomes (27), while pre-injury use of APAs increases the risk of presenting post-traumatic cerebral haemorrhage (28). This risk is increased even further with the use of second-generation APAs (clopidogrel) in individuals with mild TBI (29). Regarding the new oral anticoagulants (NOAC), the few prospective clinical studies conducted on iTBI have had conflicting results, making it difficult to draw valid conclusions (30, 31), however a retrospective study found increased hematoma expansion in individuals with pre-injury use of NOACs compared to warfarin (32). Recently published data from the Collaborative European NeuroTrauma Effectiveness Research in Traumatic Brain Injury (CENTER-TBI) corroborate the aforementioned epidemiological data, indicating that prior use of OACs/APAs increases the risk of development or progression of haemorrhagic lesions, with a 3-fold higher mortality rate and greater frequency of poor outcomes at 6 months after iTBI, especially in individuals taking vitamin K antagonists (12, 13). Mathieu et al. (33) serially and comparatively analysed CT scans in iTBI patients who used antithrombotic agents (APAs or OACs) pre-injury vs. those who did not. In the group with prior antithrombotic use, an increase in the volume of extra-axial hematomas was observed, as well as an increase in the rate of haematoma expansion and the risk of developing delayed traumatic intracerebral haemorrhages (33). A Swedish study evaluating the risk of bleeding in individuals with prior use of OACs vs. APAs and iTBI secondary to ground-level falls found an increased risk of bleeding in the group that had used APAs (34). Finally, a recent meta-analysis (low quality of the included studies) that sought to establish the adverse effects of pre-iTBI APA use, found associations between dual therapy and the progression of lesions (OR 2.81; 95% CI 1.19–6.65; *p* = 002) and need for neurosurgery (OR 1.61; 95% CI 1.15–2.28) but no related impact on hospital mortality (35).

### 3.3 Factor 3: arterial blood pressure (hypotension) was defined by participants as “the most dreaded latent threat for the traumatised brain”

Maintaining cerebral blood flow (CBF), one of the primary goals in the management of TBI, is usually associated with the metabolic rate of oxygen (CMRO_2_) (1, 4, 17). Its key determinants are cerebral perfusion pressure (CPP) and the diameter of resistance arterioles (50–150 μm) (1, 4). CPP is the result of the difference between mean arterial pressure (MAP) and intracranial pressure (ICP) (1, 4).


CPP=MAP−ICP

The CBF remains normal and stable through the intrinsic capacity of its resistance vessels to modify their diameter, a phenomenon called “cerebral autoregulation” (CAR). The change in diameter is due to different stimuli, including MAP, arterial partial pressure of oxygen (PaO_2_), arterial partial pressure of carbon dioxide (PaCO_2_), and others of neural origin (1). CAR is a natural survival mechanism that is not infinite; on the contrary, it typically functions within certain CPP levels ranging from 50 to 150 mmHg. Above or below these limits, the CBF passively follows changes in MAP (1, 17).

During TBI, however, this mechanism is altered, and the limits narrow and shift to the right. Therefore, during injury, higher levels of CPP are necessary to maintain adequate levels of CBF (17, 21). When CAR is disrupted or completely lost, even temporarily, arterial hypotension is deleterious as it causes a decrease in CBF and ischemic hypoxia with irreversible and devastating consequences (1, 17).

The traumatised brain does not cause arterial hypotension *per se* and it should not always be attributed to hypovolaemia, which is why its cause should be thoroughly investigated (36). Arterial hypotension independently increases the possibility of poor outcomes (37, 38) and is a risk factor for the development of C-iTBI (12, 13).

### 3.4 Factor 4: goal-directed haemostatic therapy was defined by participants as a factor in which “Knowledge of pathophysiology is essential”

Goal-directed haemostatic therapy (GDHT) is an element of personalised precision medicine. This approach focuses on the specific point problem a given pathology can cause, considering the overall context at all times. In order to do so, monitoring systems capable of promptly alerting healthcare professionals to any alterations that threaten physiological homeostasis are essential. At the same time, in-depth knowledge of the pathophysiology of the disorder is necessary for the correct interpretation, analysis, and decision-making based on the information such monitoring provides.

The coagulation system is finely tuned process, continuously balancing the mechanisms that promote and prevent clot formation. C-iTBI, a multifactorial entity different from coagulopathy secondary to multisystem trauma without TBI, occurs when these mechanisms are disrupted (6–10, 39). While its pathophysiology has yet to be accurately defined, C-iTBI generally begins within 24 h of admission; the more severe the TBI, the earlier it manifests (40). Its duration is variable, averaging 72 h, although this period is sometimes prolonged (40). C-iTBI is characterised by an initial hypocoagulable state consisting of platelet dysfunction, increased consumption of platelets and coagulation factors, disseminated intravascular coagulation (DIC), and hyperfibrinolysis, followed by a hypercoagulable, prothrombotic state, both local (cerebral microcirculation-ischemic lesions) and systemic (deep vein thrombosis). However, these states are linked and often it is difficult to differentiate one from the other (6–10, 39).

Although discussion of these mechanisms is beyond the scope of this manuscript, it is worth noting that multiple mediators and cascades are involved in the genesis of C-iTBI, including protein C, protein S, thromboxane, prostaglandins, adenosine, and brain tissue factor (6, 10, 39).

### 3.5 Factor 5: use fluids cautiously was defined by participants as a factor in which “Type and volume matter”

Fluid administration is one of the key elements in the initial resuscitation of TBI, especially when necessary to correct arterial hypotension and maintain adequate CPP values (41). Recent guidelines recommend 0.9% normal saline as the fluid of choice (41). Some considerations are necessary in this context such as avoiding hypotonic solutions (dextrose 5%, lactated ringer, albumin 4%) and the use of colloids (41). In terms of the implications that fluids have on coagulation, both isotonic crystalloids and colloid solutions provoke haemodilution, causing a decrease in the function and number of platelets and plasma factors involved in coagulation (42–44). This effect is directly proportional to the volume infused (42–44). Available evidence indicates that 0.9% saline at low doses does not affect coagulation (45) or has a minimal hypercoagulable effect, while at higher doses the effect is the opposite (hypocoagulable), associated with increased bleeding (42–44, 46–49). Infusions of more than 2,000 mL of fluids have been identified as an independent risk factor for the development of C-iTBI (51). Because the osmotically active solutions used for the control of intracranial hypertension such as hypertonic saline or mannitol are crystalloids, they retain their dose-dependent haemodilution capacity and cause hypocoagulable states (42–44). However, clinical studies have shown that neither 20% mannitol nor 3.5% saline affect coagulation at equimolar doses and limited infusion volumes during neurosurgery or in moderate TBI (50, 51). On the other hand, all colloids (dextran, gelatins, and hydroxyethyl starches) invariably affect coagulation, compromising platelet function and fibrin formation, decreasing the activity of coagulation factors, and increasing fibrinolysis (42–44).

### 3.6 Factor 6: low calcium levels was defined by participants as a “necessary and essential co-factor”

Calcium (Ca++) is an essential co-factor for the enzymatic activation of the coagulation cascade, specifically K- Vitamin dependent factors (II, VII, IX, and X) and factor XIII, which is involved in the structure and strength of the clot (52–54). Additionally, it plays a central role in platelet activation (52–54). Eleven percent of individuals suffering from spontaneous intracerebral haemorrhage in the acute phase present hypocalcaemia (serum Ca++ < 8.4 mg/dL), which is associated with the development of coagulopathy, higher haematoma volume, and increased risk of haematoma expansion (55). Shock associated with polytrauma (without TBI), causes a decrease in ionised Ca++ (<1.1 mmol/L) in 50% of individuals, a condition associated with the development of coagulopathy, increased transfusion requirements, and death (55), findings corroborated in a recent systematic review (56). In patients with moderate and severe TBI, decreases in ionic Ca++ (<1.1 mmol/L) on the third day postadmission were found to be predictive of mortality and poor functional outcomes (57). The main pathophysiological reasons for the decrease in calcium are chelation phenomena, primarily through the action of inflammatory mediators, lactate, and intracellular proteins released by astrocytes or damaged neurons (58). Additional mechanisms of hypocalcaemia include hypoalbuminemia secondary to increased capillary permeability post-shock resuscitation and citrate chelation after transfusion of storage blood (56, 57, 59).

### 3.7 Factor 7: anaemia-transfusion was defined by participants as “Sometimes beneficial, sometimes dangerous”

Traumatic brain injury itself rarely causes blood loss requiring transfusion (60, 61). Most TBI patients are not severely anaemic at the time they are admitted to the ICU but develop anaemia during their stay (60, 61). Post-ICU anaemia is a secondary complication but its effect on outcome remains controversial (60–62). Since the landmark work of Hébert et al. (63) more than 20 years ago, the trend has been towards restrictive transfusion therapy (64, 65). There is no cutoff point to consider a haemoglobin (Hgb) level as optimal (61, 66). Inasmuch as red blood transfusion has been associated with poor outcomes after TBI (65–70), transfusion should be guided by clinical goals rather than a “magic” number (61, 64, 65).

Indications for transfusion in the context of iTBI should therefore follow the hemodynamic status, cerebral tissue oxygenation, and premorbid state (cardiopathic patients are less tolerant to anaemia) (61, 64, 65). Hgb is responsible for nearly all the O_2_ transported by the blood, provided it is of good quality and in sufficient quantity. When these conditions are compromised, cerebral tissue hypoxia (anaemic hypoxia) may develop (71, 72). Physiologically, below 7 g/dL, O_2_ transport capacity drops by half, while above 12 g/dL, O_2_ availability provides no beneficial changes; on the contrary, O_2_ transport may decrease due to increased blood viscosity and decreased CBF (61, 71). Transfusion does not ensure correction of cerebral tissue hypoxia, which is multifactorial and requires exhaustive analysis (71, 73). Blood stored for long periods decreases its 2,3-diphosphoglycerate component, which increases the affinity of Hgb for O_2_, restricting the availability of O_2_ to the cell (65–69, 74). Transfusions can generate acute lung injury (TRALI), multiorgan dysfunction, and C-iTBI depending mainly on the volume (> risk if massive) and storage time, the so-called “blood bank coagulopathy,” which causes a series of physiological alterations such as tissue hypoxia, hypocalcaemia (citrate intoxication), hypothermia, acidosis, hyperglycaemia and hyperkalaemia, generating a vicious circle that perpetuates and exacerbates C-iTBI. In sum, balancing the risks and benefits of transfusion is paramount (65, 67–69, 74). Available evidence suggests that it is reasonable to maintain Hgb values between 7 and 9 g/dL (61, 65, 66).

### 3.8 Factor 8: temperature was defined by participants as a factor where “Too low or too high, both are detrimental”

Post-trauma and shock hypothermia affect the coagulation system, resulting in an independent risk factor for mortality and poor outcomes (75–77). Hypothermia increases the affinity of Hgb for O_2_ (shift to the left), making it more difficult to transfer the necessary O_2_ to the cell (high-affinity hypoxia) (72, 78). Hypothermia generates a hypocoagulable state, facilitating bleeding by blocking coagulation enzymatic cascades, prolonging the initiation phase, altering platelet aggregability, and decreasing fibrinogen synthesis (79–82). These mechanisms are mediated by thrombin (79, 80). Hyperthermia is prevalent after TBI (83–85). During the first hours of evolution, this constitutes a secondary insult, associated with the severity of the injury and with poor outcomes (83–85). Hyperthermia increases ICP, exacerbates oedema and inflammation, causes rupture of the blood–brain barrier (BBB), and can trigger cerebral hypoxia (83–85). Temperatures between 38 and 39 degrees Celsius generally either do not modify coagulation or cause a prothrombotic state without major consequences (86), but in cases of extreme elevation (heatstroke), the fibrinolytic mechanism initially ceases to function to then consume coagulation factors and platelets, leading to DIC and multiple organ dysfunction (87).

### 3.9 Factor 9: international normalised ratio-monitoring was defined by participants as a factor in which “Interpreting information is key”

For years and through today, in ICUs worldwide, coagulation monitoring has been based on the determination of conventional coagulation assays (CCA) such as International Normalised Ratio (INR), platelet count, activated partial thromboplastin time (aPPT), and D-dimer, which have also been used to define C-iTBI (Table 1) (6–10, 12, 88).

**Table 1:** Conventional coagulation assays (CCA).

CCA	Evaluation	Cohort value to C-iTBI definition
Platelet count	Number only	< 100,000/mm (11)
INR	Prothrombin time	>1,2
PT	Time to clot formation in blood. Extrinsic pathway (old coagulation physiology)	>15 s
Thrombin time	Similar to PT	>20 s
aPPT	Contact activation in plasma	>35 s
	Heparin-sensitive	
	Intrinsic pathway	
Fibrinogen		<1.5 gr/L
D-dimer	Fibrinolysis. Unspecific	>0.5 μg/mL
Fibrinogen degradation products	Fibrinolysis	>11 mg/mL

Two issues are important to highlight. On the one hand, the methods of analysis and cohort points from which to define alterations have been variable and heterogeneous. On the other, multiple limitations emerge in assessing the role of the components during the different phases of coagulation, among which we found (6–10, 12, 88):

- Non-standardised parameters and cutoff points
- Do not assess the onset of clot formation
- Do not assess clot strength and integrity
- Low sensitivity especially in trauma
- Do not assess platelet function
- Non-specific and inaccurate assessment of the fibrinolytic system
- Do not detect the broad spectrum of clotting alterations when antithrombotic drugs were used pre-injury

**Figure 2:**
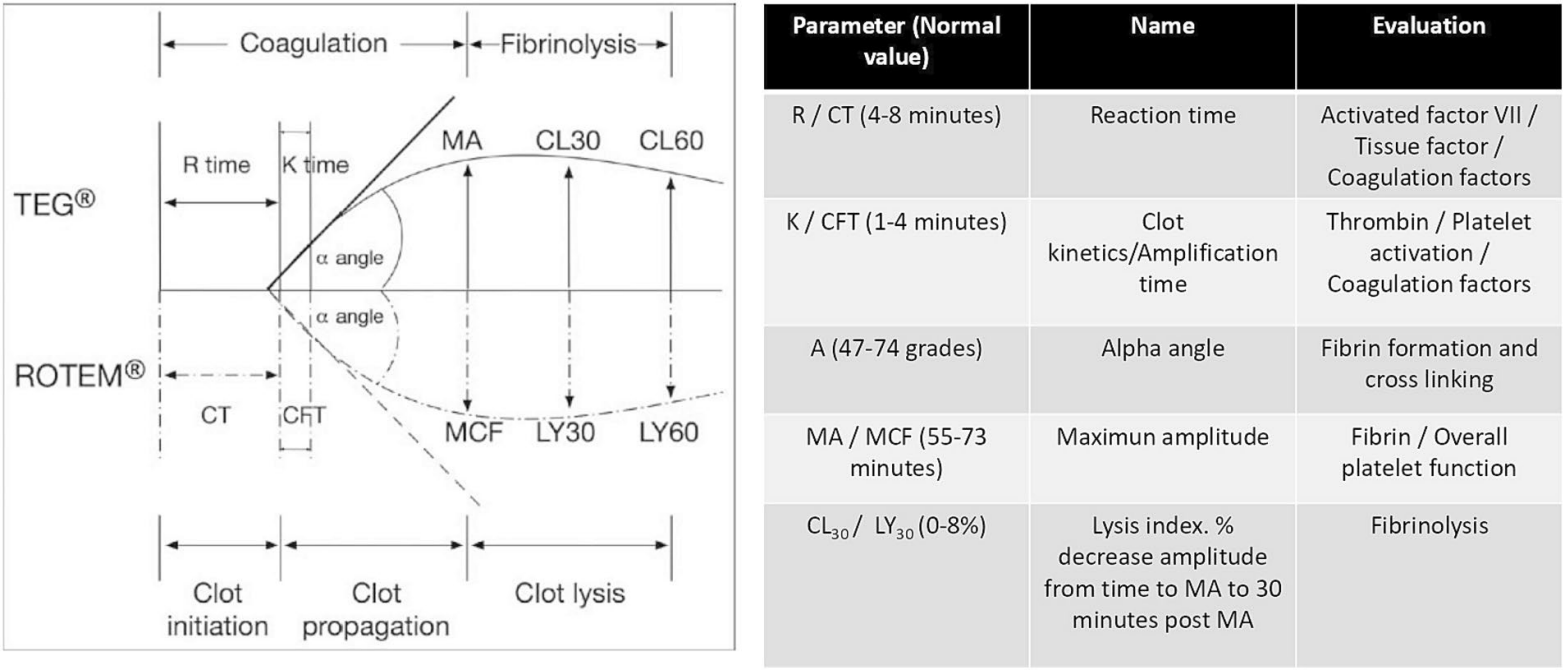
Parameters evaluated with VET.

As a point of comparison, viscoelastic tests (VET) such as TEG thromboelastography or ROTEM thromboelastometry and their variations allow for a more detailed and rapid evaluation of the full spectrum of coagulation physiology at the bedside using whole blood (6–10, 12, 88). Clot formation kinetics, fibrin-platelet interactions, platelet functionality, and fibrinolysis can be graphically monitored (6–10, 12, 88). The parameters evaluated with VETs and their cohort points reported in the literature are outlined in Figure 2.

Viscoelastic tests are more sensitive than CCAs for the full evaluation of the coagulation system (6–10, 12, 87) and their use is recommended in current guidelines for the management of bleeding secondary to trauma (89); however, these modalities also have limitations, including the following:

- Not widely available
- Longer learning curve
- They are *in vitro* tests (like CCA) that do not allow for assessment of endothelial contribution or von Willebrand factor deficiency.
- Low sensitivity for detecting abnormalities when using low molecular weight heparins or the new oral anticoagulants
- High negative predictive power, but low positive predictive power
- Quality control and maintenance of difficult systems

**Figure 3:**
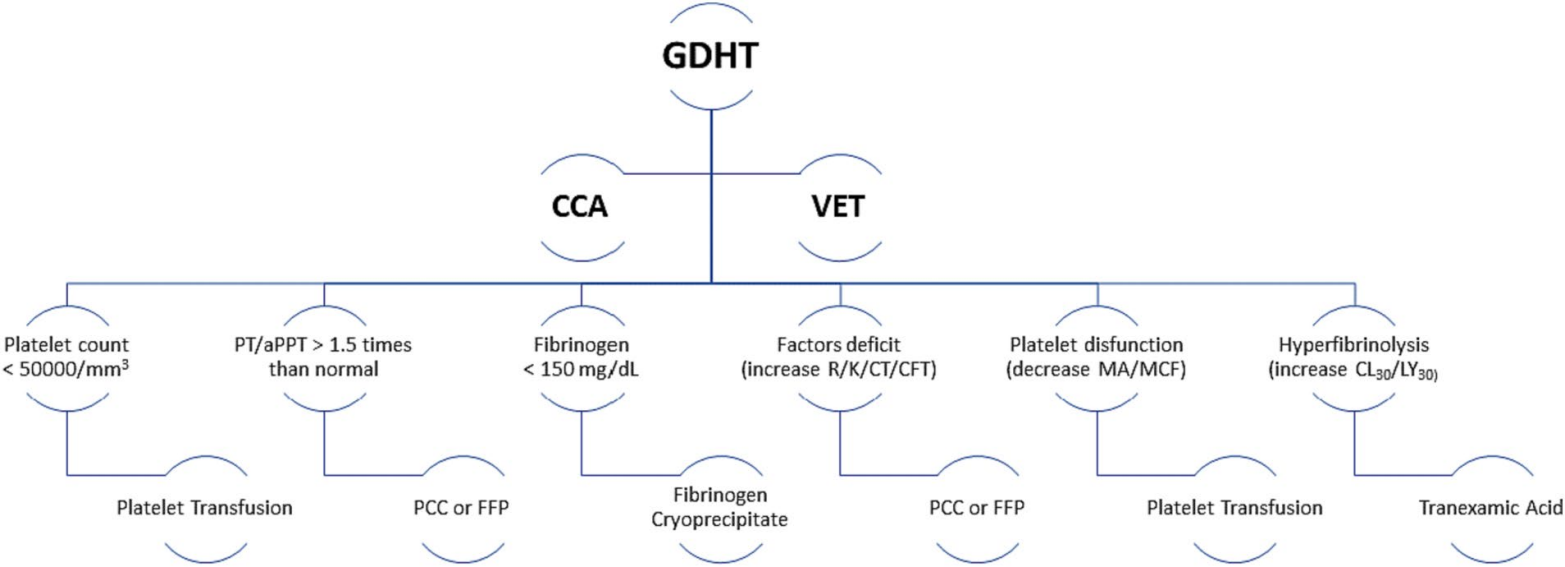
Monitoring methodology. PCC, Prothrombin complex concentrate; FFP, Fresh frozen plasma; GDHT, Goal-directed haemostatic therapy; CCA, Conventional coagulation assays; VET, Viscoelastic tests.

Coagulation disorder therapy guided by VETs following craniotomy has shown promising results (90). A recent clinical study comparing the management of coagulation disorders during trauma resuscitation demonstrated improved survival and less use of plasma and platelets in patients managed by GDHT directed by VETs vs. GHDT guided by CCA, although an analysis of the subgroup of individuals with TBI found no difference (91). Similar results were observed in the ITACTIC trial, specifically in terms of mortality (92). In the pre-defined TBI group, 64% of individuals managed and guided by VETs did not undergo massive transfusion protocols during the first day of trauma compared to 40% of patients in the group whose therapy was CCA-guided (OR 2.12, 95% CI 0.84–5.34) (91). Irrespective of the monitoring methodology available, we can direct therapy more precisely based on the results obtained. An example found in the evidence is outlined in Figure 3.

### 3.10 Factor 10: oral antithrombotic reversal was defined by participants as “Urgent, emergent, or both?”

We have previously explained why clinicians must be on “high alert” when a patient with TBI has a history of pre-injury antithrombotic drugs (12, 28–35). Due to the risk of lesion progression, and that of haemorrhagic lesions in particular, and given its close association with poor outcomes, this is a situation that requires prompt resolution (12, 28–35). Once the administration of the causative agent has been suspended, this next step must emphasise the assessment and clinical-tomographic categorisation of the TBI, establishing the presence of bleeding (volume, location, extension, anatomical distortions or displacements, and associated lesions); its severity and therapeutic possibilities, including the need for surgical intervention (89, 93–95). Coagulation monitoring (CCA or VET) is essential to establish the starting point, follow-up, and above all to avoid the undesirable effects of “overtreatment” (89). Undoubtedly, severe life-threatening bleeding associated with pre-injury use of antithrombotic agents requires immediate reversal (89, 94). In other situations, the need for reversal must be balanced against the risks involved (thrombosis) (89, 94). A detailed analysis of how and with what agents this reversal should be implemented is beyond the scope of this manuscript; but generally the strategy depends fundamentally on the antithrombotic agent in question (89, 93–97). In Table 2, we summarise the agents used in accordance with current guidelines (89).

**Table 2:** Summary of agents employed for reversal of anti-thrombotic drugs.

Antithrombotic	Reversal agent	Evidence quality
Oral anticoagulants (K-vitamin dependents)	PCC + vitamin K	1A
	If PCC is not available, FFP is an alternative	
New oral anticoagulants factor Xa inhibitors (apixaban, endoxaban, rivaroxaban)^*^ Thrombin inhibitor (dabigatran)^**^	PCC + Tranexamic Acid + Activated charcoal^***^	2C
	PCC + Idarucizumab + Activated charcoal^***^	2C + 1B
Antiplatelet agents (aspirin, dipyridamole, ticlopidine, cilostazol, clopidogrel)	Platelet transfusion^****^	2C
	Desmopressin^*****^	2C
Unfractionated heparin (UFH)	Protamine sulphate	Strong recommendation, moderate quality of evidence^***^
Low weight molecular heparin (LWMH)	Protamine sulphate	Strong recommendation, moderate quality of evidence^***^

PCC, Prothrombin complex concentrate; FFP, Fresh frozen plasma. ^*^Guidelines suggest plasma dosing of the drugs in question, calibrating the anti-factor Xa activity for each agent. Consult with Haematologist (Level of evidence 2C) (89). ^**^Guidelines suggest plasma dosing of dabigatran. If not available, follow up with thrombin time. Consult with Haematologist (Level of evidence 2C) (89). ^***^Taken from reference (94). ^****^Only for individuals with active bleeding and demonstrated evidence of platelet dysfunction (2C) or undergoing surgery (2B) (89). ^*****^Suggested for individuals with pre-injury intake of antithrombotic agents or von Willebrand disease (89).

Although they are not oral agents, we have included unfractionated heparins (UFH) and low molecular weight heparins (LWMH) here due to their frequent pre-injury use, particularly in older adult with significant prothrombotic comorbidities or undergoing prolonged postoperative periods, especially orthopaedic surgery (94).

### 3.11 Factor 11: normal acid–base status was defined by participants as “Neither acid nor alkaline; neutral”

During acute TBI it is essential to achieve a balanced physiological microenvironment to prevent secondary insults (1, 4, 36, 71). Acidosis causes cerebral vasodilation, increased cerebral blood volume, and consequent increase in ICP; while alkalosis increases the affinity of Hgb for O_2_ without the possibility of ceding it to the cells, causing cerebral tissue hypoxia of low extraction (1, 4, 36, 71, 72). Coagulation is not exempt from modifications if the acid–base state is modified (75, 76, 80). Although alkalosis does not modify coagulation parameters (86); acidosis compromises both platelet aggregation and clot formation, increases the prolongation phase due to alterations in thrombin, and accelerates the degradation of fibrinogen (75–77).

## 4 Summary of the design and implications of the mnemonic device “Coagulation”

In this study, we complemented our narrative analysis with evidence-based studies. The methodological premise was that analytic actions should coincide with both evidence-based studies and the characteristics of the factor identified by study participants.

**Figure 4:**
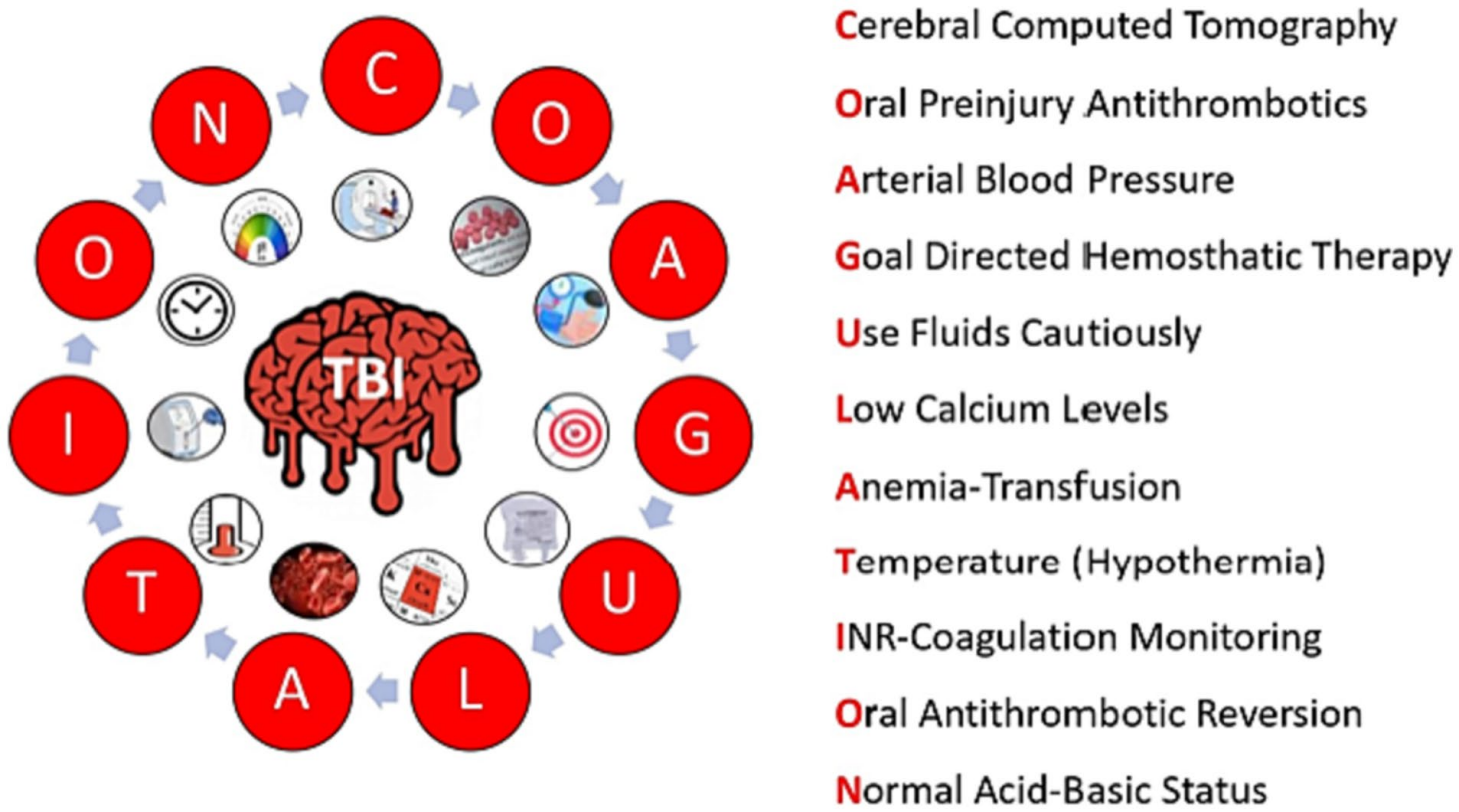
The mnemonic device “Coagulation.”

In the Figure 4, we summarise the algorithm based on the comprehensive analysis of the acronym “Coagulation,” we designed as a mnemonic device encompassing the primary factors involved in the management of C-iTBI in the intensive care unit. In the process, we have suggested a comprehensive clinical approach to the variables and situations that may predispose patients to developing this condition, as well as the management of risk factors and complications requiring prompt resolution.

## 5 Conclusion

Narrative analysis is an interdisciplinary tool that may be applied as a qualitative approach to research using temporally situated data from a wide range of discourses. In this study, we conducted a narrative analysis based on the responses of intensive care physicians regarding the key factors associated with C-iTBI.

Knowledge and detection of the risk factors, pathophysiology, and kinetics of the coagulation system and its alterations allow for the prompt recognition and management of traumatic injury to the brain. The essential information obtained from the serial monitoring of coagulation in conjunction with clinical judgement allows for a personalised and goal-directed therapy that can help prevent or minimise the adverse effects of conventional therapies and reduce the negative consequences of coagulopathy.

Given the time-sensitive nature of such decisions, in this study, we aimed to design an acronym as a mnemonic device to provide clinicians with a simple auxiliary tool in the treatment of this critical complication. We believe that the acronym “Coagulation” meets the stated objectives. It is a simple and easy to apply tool for anyone facing the challenge of treating victims of moderate or severe TBI on a daily basis.

Numerous challenges remain to prevent or minimise secondary damage and ensure improved patient outcomes in C-iTBI. Issues such as the harmonisation of definitions and validation of timely, appropriate, personalised, goal-based management require the prompt design and execution of large-scale clinical studies. The dissemination of knowledge as well as technological advances in the monitoring systems of the coagulation system offer a unique opportunity to unify criteria aimed at achieving increasingly precise medicine in this critical field.

## 6 Limitations and bias

This study is presented as a narrative method supported by scientific evidence and must take into account the limitations that such an approach entails. It is also specifically focused on TBI.

## 7 Clinical implications

In this article, we provide a mnemonic device for critical situations that will improve clinical care in the context of intensive care units.

## Data availability statement

The raw data supporting the conclusions of this article will be made available upon request to the last author.

## Author contributions

MQ-D: Conceptualization, Data curation, Formal Analysis, Funding acquisition, Investigation, Methodology, Project administration, Resources, Writing – original draft, Writing – review & editing, Supervision, Validation. PA: Investigation, Methodology, Project administration, Writing – original draft. RJ-V: Methodology, Project administration, Resources, Writing – original draft. EE-S: Software, Validation, Writing – original draft. CT-G: Data curation, Formal Analysis, Investigation, Writing – review & editing. KN-N: Software, Validation, Writing – original draft. AS-L: Methodology, Project administration, Writing – original draft. PM-N: Validation, Visualization, Writing – original draft. MG-E: Resources, Validation, Visualization, Writing – review & editing. JG-C: Methodology, Project administration, Validation, Visualization, Writing – review & editing. DG: Supervision, Writing – original draft, Writing – review & editing. PS-C: Software, Writing – original draft.
